# P18 Antimicrobial resistance patterns of *Campylobacter* species isolated from poultry in Mathira, Nyeri County, Kenya

**DOI:** 10.1093/jacamr/dlac053.018

**Published:** 2022-05-31

**Authors:** Edith Chege

**Affiliations:** National Veterinary Laboratories, Nairobi, Kenya

## Abstract

**Background:**

*Campylobacter* species are zoonotic pathogens recognized as a cause of gastroenteritis in the human population and pose a major public health threat worldwide. The most important in the genus is *Campylobacter jejuni*, which causes diarrhoea in patients, mainly children under the age of 1 year, young adults and immunosuppressed persons. *Campylobacter* occurs in the gut of most animals as normal flora and is transferred to foods from the faeces during slaughter. It is acquired through consumption of undercooked poultry and other meat as well as water and unpasteurized milk. Fruits and vegetables can be contaminated from water containing faeces from birds and other animals. *Campylobacter* can also be transmitted through contact with dogs and cats’ faeces as well as drinking contaminated water. *Campylobacter* infections are common in low- and middle-income countries as well as developed countries.

**Objectives:**

To assess the prevalence and determine the antimicrobial resistance patterns of *Campylobacter* spp. from poultry in Mathira Sub-County, Nyeri County.

**Methods:**

A total of 380 cloacal swabs were collected randomly form 57 farmers in Mathira, Kenya. They were transported in sterile Amies charcoal swabs under cold chain and stored at 4°C. They were enriched in Preston broth and incubated for 24 h at 42°C, after which they were streaked onto mCCDA agar and incubated for 24 h at 42°C under microaerobic conditions generated by Campy Gen^™^ packs. Typical grey moist, swarming and discrete colonies were identified using MALDI-TOF. Antimicrobial susceptibility testing was done on ampicillin, tetracycline, ciprofloxacin, gentamicin, erythromycin and nalidixic acid by the disc diffusion method and disc diameters were measured and interpreted using the CLSI guidelines.

**Results:**

In total, 272 out of 380 (71.32%) isolates were *Campylobacter*; 190 (50%) were *C. jejuni* while 81 (22) were *Campylobacter coli*. Resistance to ampicillin was 40% for *C. coli* and 30.53% for *C. jejuni*. Overall, 53.75% of *C. coli* isolates and 51.85% of *C. jejuni* isolates were resistant to tetracycline. We found 67.50% of *C. coli* were resistant to ciprofloxacin as compared with 38.42% of *C. jejuni*. Resistance to erythromycin for *C. coli* and *C. jejuni* was 10% and 16.935%, respectively. Finally, 65% of *C. coli* was resistant to nalidixic acid as compared with 38.42% of *C. jejuni*.

**Conclusions:**

The study found that *Campylobacter* spp. show high levels of resistance to tetracyclines, ciprofloxacin and nalidixic acid and are MDR. Resistance to gentamicin and erythromycin is markedly low and these antibiotics can be reserved for treatment. Surveillance activities for AMR should be enhanced and laboratory capacity increased to support surveillance and diagnosis.

